# State-Based Medicaid Costs for Pediatric Asthma Emergency Department Visits

**DOI:** 10.5888/pcd11.140139

**Published:** 2014-06-26

**Authors:** William S. Pearson, Scott A. Goates, Samantha D. Harrykissoon, Scott A. Miller

**Affiliations:** Author Affiliations: Scott A. Goates, Samantha D. Harrykissoon, Scott A. Miller, Centers for Disease Control and Prevention, Atlanta, Georgia.

## Abstract

**Introduction:**

The prevalence of childhood asthma in the United States increased from 8.7% in 2001 to 9.5% in 2011. This increased prevalence adds to the costs incurred by state Medicaid programs. We provide state-based cost estimates of pediatric asthma emergency department (ED) visits and highlight an opportunity for states to reduce these costs through a recently changed Centers for Medicare and Medicaid Services (CMS) regulation.

**Methods:**

We used a cross-sectional design across multiple data sets to produce state-based cost estimates for asthma-related ED visits among children younger than 18, where Medicaid/CHIP (Children’s Health Insurance Program) was the primary payer.

**Results:**

There were approximately 629,000 ED visits for pediatric asthma for Medicaid/CHIP enrollees, which cost $272 million in 2010. The average cost per visit was $433. Costs ranged from $282,000 in Alaska to more than $25 million in California.

**Conclusions:**

Costs to states for pediatric asthma ED visits vary widely. Effective January 1, 2014, the CMS rule expanded which type of providers can be reimbursed for providing preventive services to Medicaid/CHIP beneficiaries. This rule change, in combination with existing flexibility for states to define practice setting, allows state Medicaid programs to reimburse for asthma interventions that use nontraditional providers (such as community health workers or certified asthma educators) in a nonclinical setting, as long as the service was initially recommended by a physician or other licensed practitioner. The rule change may help states reduce Medicaid costs of asthma treatment and the severity of pediatric asthma.

## Introduction

Asthma is a chronic respiratory disease characterized by reversible periodic airway obstructions initiated by certain exposures, including environmental hazards (1). The disease is becoming increasingly prevalent in the United States, particularly among children and certain minority groups. More than 25 million people are currently diagnosed with the disease, including 6.5 million children younger than 18 (2). Although no cure for asthma is available, symptoms can be controlled with appropriate medical treatment, self-management, and education, and by avoiding exposure to environmental allergens and irritants that can trigger an attack (1).

Asthma in the United States costs $56 billion annually for direct health care expenditures and indirect costs such as lost productivity (3). In 2009, asthma resulted in approximately 480,000 hospitalizations, 1.9 million emergency department (ED) visits, and nearly 9 million doctors’ office visits (4). As the third leading cause of hospitalization among children younger than 15, asthma is associated with increased ED visits (5). Additionally, studies have reported a higher rate of health care use among children insured by Medicaid compared with children insured by other health insurance programs (6).

Despite increased attention on asthma prevention and advances in diagnosis and treatment, childhood asthma prevalence in the United States has increased from 8.7% in 2001 to 9.5% in 2011 (7). Having a clinical care system that focuses on improving asthma management before cases become acute is essential. However, given the complexity of the disease, a comprehensive, community-based approach to educate and assist children with asthma outside of traditional clinical settings may be beneficial.

Asthma education programs provided in nontraditional settings, for example, in homes, can reinforce self-management education and treatment by reaching children and parents where they live. These evaluated programs follow the National Asthma Education and Prevention Program evidence-based guidelines (1) that emphasize the need for asthma education at all points of care. Some studies have demonstrated that asthma home visitation programs may improve asthma management, reduce the number of missed school and work days, decrease allergens in the home, reduce urgent care use, and lessen caregiver stress (8,9). Furthermore, in its systematic review of research, the Community Preventive Services Task Force found that home-based interventions with an environmental focus improve the overall quality of life and productivity in children with asthma (10).

Despite the significant burden of pediatric asthma ED visits, we were unable to find comprehensive estimates of Medicaid and Children’s Health Insurance Program (CHIP) costs at the state level. We analyzed the cost of pediatric asthma ED visits, which are primarily paid for by Medicaid or CHIP at a state level. We then highlighted an opportunity for states to reduce asthma costs borne by their Medicaid programs because of a recent change to a Centers for Medicare and Medicaid Services (CMS) regulation. The rule change allows Medicaid to reimburse nontraditional health care providers for providing asthma prevention services in nontraditional settings.

## Methods

Because data on pediatric asthma ED visits were not available for all states, we constructed a “second-best” approximation by using multiple sources of data that may not have included data from the same years. We chose the most recent data from each source to derive an estimate for a single year. We initially estimated the number of ED visits made by children aged 0 to 17 years where asthma was listed as one of the reasons for the visit and Medicaid or CHIP was the primary payer for the visit. For this part of the analysis, we used data from the National Hospital Ambulatory Medical Care Survey Emergency Department (NHAMCS-ED) survey.

The NHAMCS-ED is a national survey that collects information on the delivery and use of emergency care services at noninstitutional, general, and short-stay hospitals, excluding federal government facilities such as Veteran’s Administration hospitals and military hospitals in the states and the District of Columbia. This multistage design survey collects data on randomly selected patient records during a 4-week sampling period (11).

To analyze all ED visits for asthma by children younger than 18, we searched hospital records for International Classification of Diseases, 9th revision (ICD-9) codes to estimate the total visits in 2010. Each visit record contained up to 3 ICD-9 codes that contributed to the reason for the visit. We searched for the asthma code of 493.xx and its subcategories (493–493.92) among these 3 ICD-9 diagnosis codes. We further limited our search to visits for which Medicaid or CHIP was the primary payer. We considered all ED visits with any diagnosis of asthma, regardless of whether the patient was discharged home or admitted to the hospital.

State-by-state asthma prevalence data were obtained from the National Survey of Children’s Health (NSCH), 2012. This survey was sponsored by the Maternal and Child Health Bureau; the National Center for Health Statistics (NCHS) of the Centers for Disease Control and Prevention (CDC) oversaw the sampling. Data were collected by using the State and Local Area Integrated Telephone Survey (developed by NCHS) to randomly sample families from all 50 states and the District of Columbia. A total of 95,677 surveys were completed nationally for children aged 0 to 17 years, and a minimum of 1,800 surveys were collected for each state (12).

We used CMS enrollment data to determine the total number of children enrolled in state Medicaid/CHIP programs for each state. We obtained state-by-state enrollment data for children enrolled in Medicaid and CHIP in 2010 from the Kaiser Family Foundation website. The data provided in this report were available only for children aged 0 to 18 (13). We multiplied state enrollment data by the prevalence data for each state to estimate the total number of children with asthma covered by Medicaid or CHIP. This data source did contain enrollment data on children aged 18, which is 1 year older than the age range of 0 to 17 years for all other data sources used in these analyses. We were not able to adjust this number.

State estimates for the number of asthma ED visits for children younger than 18 were estimated by taking the total number of ED visits nationally (estimated by the NHAMCS-ED) and apportioning the visits to the states based on estimates of the number of Medicaid/CHIP enrollees with asthma in the state. For example, if a state covered 100,000 children under Medicaid/CHIP and had an asthma prevalence rate of 7%, we estimated that approximately 7,000 (100,000 × 7%) children with asthma were covered by Medicaid/CHIP in that state. If these 7,000 children represented 5% of all children with asthma covered nationally by Medicaid/CHIP, then we assumed that this state had 5% of all childhood asthma ED visits nationally.

We used Marketscan data to estimate the cost for pediatric asthma ED visits and subsequent hospitalizations. Costs for ED asthma visits and hospitalizations were obtained from the Marketscan Medicaid 2011 data set (the most recent year available). The Marketscan data set contains a convenience sample of claims data for Medicaid participants of 11 unnamed states (14). We estimated costs by identifying children younger than 18 who were treated in the ED with asthma as either the primary diagnosis or 1 of 3 secondary diagnoses. Cost estimates were limited to cases where Medicaid was the expected payer, and the patient was enrolled in a noncapitated plan.

## Results

According to NHAMCS-ED data, Medicaid/CHIP enrollees younger than 18 made an estimated 628,759 asthma-related ED visits in 2010. Using the Marketscan Medicaid database, we estimated that the average cost per visit was $433. Given these estimates, pediatric asthma-related ED visits cost the Medicaid/CHIP programs a combined $272,453,850 in 2010.

Seven states (California, Florida, Georgia, Illinois, New York, Pennsylvania, and Texas) are estimated to have spent more than $10 million each in 2010 on asthma-related visits for this population (Figure, Table). California has a low prevalence of asthma (6.5%) but the highest cost because it covers a large number of enrollees. Other states, such as New York (10.5%) and Pennsylvania (10.9%), have higher prevalence than states of similar size, which contribute to higher asthma costs. For perspective, if the prevalence in New York had been the same as California’s, New York would have had 16,665 fewer asthma-related ED visits for this population and saved $7.2 million in 2010.

**Figure:**
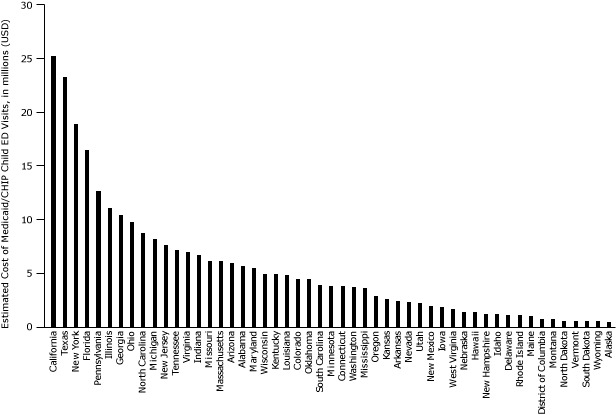
Medicaid/CHIP Spending on Asthma-Related Emergency Department Visits for Children Aged 0–17 Years, by State, 2010. Abbreviations: CHIP, Children’s Health Insurance Program; ED, emergency department. **Table:** 

State	Estimated Cost of Medicaid/CHIP Child ED Visits, $^a^
Total	272,454,000
AK	282,000
AL	5,578,000
AR	2,376,000
AZ	5,916,000
CA	25,219,000
CO	4,451,000
CT	3,751,000
DC	652,000
DE	1,031,000
FL	16,538,000
GA	10,400,000
HI	1,256,000
IA	1,762,000
ID	1,093,000
IL	11,102,000
IN	6,646,000
KS	2,519,000
KY	4,784,000
LA	4,713,000
MA	6,041,000
MD	5,422,000
ME	940,000
MI	8,219,000
MN	3,806,000
MO	6,069,000
MS	3,595,000
MT	597,000
NC	8,762,000
ND	475,000
NE	1,303,000
NH	1,115,000
NJ	7,586,000
NM	1,884,000
NV	2,221,000
NY	18,956,000
OH	9,689,000
OK	4,424,000
OR	2,785,000
PA	12,704,000
RI	1,019,000
SC	3,834,000
SD	457,000
TN	7,181,000
TX	23,214,000
UT	2,109,000
VA	6,964,000
VT	464,000
WA	3,696,000
WI	4,864,000
WV	1,601,000
WY	390,000

^a^ These estimates have been rounded to the nearest $1,000.

We estimated that 36 states had Medicaid/CHIP costs ranging from $1 million to $10 million for asthma-related pediatric ED visits. Among these states, Delaware, Alabama, Tennessee, Oklahoma, Mississippi, Kentucky, Connecticut, Rhode Island, Missouri, Massachusetts, and Louisiana had asthma prevalence above 10%. If a high-prevalence state such as Alabama (11.8%) had the same prevalence as Washington State (5.6%), Alabama could have eliminated about 6,763 ED visits for this population and saved $2.9 million in 2010.

Seven states and the District of Columbia had estimated Medicaid/CHIP costs under $1 million for asthma-related pediatric ED visits. Five (North Dakota, Montana, Alaska, South Dakota, and Wyoming) had low asthma prevalence (<8%) and fewer enrollees compared with other states. In contrast, the District of Columbia had a high asthma prevalence of 15.5%, and Vermont had an asthma prevalence of 8.6%. If the asthma prevalence for Vermont equaled that for South Dakota, Vermont could have eliminated 398 ED visits for this population and saved $172,602 in 2010 (Figure, Table).

**Table:** Estimates of Asthma Prevalence, Medicaid/Children’s Health Insurance Program (CHIP) Enrollment, and Asthma-Related Pediatric Emergency Department (ED) Visits by State for US Children Aged 0–17 Years, 2010

State	Estimated Asthma Prevalence Among Children, %	Estimated No. of Children Covered by Medicaid/CHIP	Estimated No. of Medicaid/CHIP-Covered Children with Asthma^a^	Estimated No. of Asthma-Related ED Visits Covered by Medicaid/CHIP	Estimated Cost of Medicaid/CHIP Child ED Visits ($)^b^
Total	8.8	37,615,535	3,313,869	628,759	272,454,000
AK	3.6	85,003	3,060	650	282,000
AL	11.8	585,768	69,121	12,873	5,578,000
AR	8.0	455,683	36,455	5,483	2,376,000
AZ	8.7	720,427	62,677	13,652	5,916,000
CA	6.5	5,614,747	364,959	58,200	25,219,000
CO	8.7	444,142	38,640	10,271	4,451,000
CT	11.0	330,310	36,334	8,657	3,751,000
DC	15.5	88,179	13,667	1,505	652,000
DE	12.0	98,380	11,805	2,379	1,031,000
FL	9.9	2,151,703	213,019	38,166	16,538,000
GA	10.0	1,321,941	132,194	24,001	10,400,000
HI	9.9	137,057	13,569	2,897	1,256,000
IA	5.8	315,976	18,326	4,067	1,762,000
ID	6.1	166,424	10,152	2,521	1,093,000
IL	8.5	1,738,547	147,776	25,622	11,102,000
IN	9.9	765,624	75,797	15,338	6,646,000
KS	8.3	264,408	21,946	5,812	2,519,000
KY	11.2	507,771	56,870	11,041	4,784,000
LA	10.1	765,634	77,329	10,877	4,713,000
MA	10.2	635,442	64,815	13,942	6,041,000
MD	9.6	574,712	55,172	12,512	5,422,000
ME	8.2	141,526	11,605	2,169	940,000
MI	8.4	1,219,772	102,461	18,967	8,219,000
MN	7.1	451,077	32,026	8,782	3,806,000
MO	10.2	654,024	66,710	14,006	6,069,000
MS	11.4	469,395	53,511	8,297	3,595,000
MT	6.4	96,432	6,172	1,378	597,000
NC	9.2	1,132,557	104,195	20,221	8,762,000
ND	7.6	48,808	3,709	1,097	475,000
NE	6.8	188,248	12,801	3,008	1,303,000
NH	9.3	107,748	10,021	2,573	1,115,000
NJ	8.8	742,003	65,296	17,507	7,586,000
NM	8.7	358,242	31,167	4,347	1,884,000
NV	8.0	224,013	17,921	5,125	2,221,000
NY	10.5	2,489,543	261,402	43,745	18,956,000
OH	8.5	1,335,574	113,524	22,359	9,689,000
OK	11.4	559,482	63,781	10,209	4,424,000
OR	7.7	380,566	29,304	6,427	2,785,000
PA	10.9	1,274,545	138,925	29,317	12,704,000
RI	10.9	112,099	12,219	2,352	1,019,000
SC	8.5	540,188	45,916	8,847	3,834,000
SD	5.4	92,841	5,013	1,055	457,000
TN	11.5	860,553	98,964	16,573	7,181,000
TX	8.1	3,699,623	299,669	53,572	23,214,000
UT	5.8	246,406	14,292	4,867	2,109,000
VA	9.0	671,413	60,427	16,071	6,964,000
VT	8.6	72,805	6,261	1,071	464,000
WA	5.6	796,010	44,577	8,531	3,696,000
WI	8.7	596,145	51,865	11,226	4,864,000
WV	9.9	223,847	22,161	3,695	1,601,000
WY	6.9	62,172	4,290	900	390,000

a Childhood asthma prevalence is the weighted average of each state’s asthma prevalence total weighted by the number of covered children in the state and is not a nationally representative prevalence estimate.

b These estimates have been rounded to the nearest $1,000.

## Discussion

Our analysis estimated that state Medicaid programs combined spent in excess of $272 million on pediatric asthma-related ED visits in the United States in 2010. These results supplement data presented in the CDC Chronic Disease Calculator that provides estimates of the overall costs of asthma to states. Our data focuses specifically on pediatric ED visits, which are only a small portion of total asthma costs (15).

This research addresses a gap in the literature pertaining to Medicaid expenditures associated with pediatric asthma ED visits, costs that can be reduced through increased access to evidence-based interventions, including those provided in the home. To our knowledge, these estimates are the first of their type and could help state Medicaid directors and policy makers determine policy options for reducing costs of pediatric asthma.

Expanded coverage into nontraditional settings resulting in an uptake of preventive services could reduce asthma prevalence and asthma health disparities. Further research will better quantify whether reduced asthma-related costs result from nontraditional providers delivering services in home-based settings.

Home-based interventions can show a return on investment. The Community Preventive Services Task Force documented studies that demonstrated savings ranging from $5.30 to $14 for every dollar invested in home-based asthma interventions focused on children and adolescents. Community-based asthma interventions, including those provided in the home, help children proactively mitigate asthma triggers and help them manage asthma symptoms throughout their daily routines (16). A study of Boston’s Community Asthma Initiative (CAI) indicated that the program involving 283 children — which included home-based asthma education and other asthma services provided by community health workers — improved health outcomes and reduced ED visits, hospital admissions, missed school days, and parent work days (17). A related analysis found that “investments in a community asthma case management program can lead to large returns from the cost savings generated by the program” and that the CAI program was paying for itself within 2 years of implementation and was demonstrating cost savings after 3 years (18).

Innovative research projects being conducted by the New England Asthma Innovations Collaborative and funded by the Centers for Medicare and Medicaid Innovation (CMMI) are focusing on service delivery and payment systems for asthma. Those projects may also contribute to the evidence base for providing and paying for services that prevent asthma. That project is novel in exploring the spectrum of asthma preventive services in various settings, which are provided by both nonclinicians and clinicians and paid for by public or private insurance plans (19). Additional innovative projects would help us understand how payment mechanisms can influence health outcomes.

Effective January 1, 2014, CMS implemented an updated regulation (42 CFR section 440.130) regarding which providers can be reimbursed for providing preventive services to Medicaid and CHIP beneficiaries. Prior to January 1, 2014, the regulation limited the coverage of preventable services to those that were actually provided by a physician or other licensed practitioner. As a result, some state Medicaid programs limited their coverage of preventive services to those furnished by licensed providers in a clinical setting. The regulation subsequently limited access to services and evidence-based interventions in homes and other community settings for Medicaid beneficiaries. In the final rule, CMS updated the regulation to allow state Medicaid programs to reimburse for preventive services provided by professionals such as community health workers or asthma educators so long as the service was initially recommended by a physician or other licensed practitioner (20). This change adds more flexibility to the Medicaid regulation that already allows states discretion over the setting in which care is provided. Thus, this final rule change on provider qualification, in combination with existing flexibility for states to define practice setting, can allow states to reimburse for asthma interventions that use nontraditional providers in a nonclinical setting.

Our estimates of asthma-related state ED visits covered by Medicaid and CHIP are subject to several limitations. One limitation is the different definition of “child” in different data sets used for our analysis. NSCH defines a child as being younger than 18 years, whereas our estimates of child enrollment in state Medicaid/CHIP programs include 18-year-olds. Provided that the proportion of 18-year-old enrollees relative to the number of total childhood enrollees is similar between states, our estimates are unbiased. If a particular state has a larger number of 18-year-olds, the burden estimate for that state will be overestimated, and the burden estimate for other states will be underestimated. Our estimates also assume that the ED visit rate per childhood asthma enrollee is constant among states. If the ED visit rate is lower in a particular state, then that state’s burden will be overestimated by our methods and other states’ burden would be underestimated.

Another limitation is that we identified our sample of visits by including the ICD code of 493.xx in any of the 3 possible codes related to the visit. Our approach may overestimate the numbers of visits directly caused by asthma. However, asthma was listed as a contributing factor to the visit. The clinician may also use a different ICD code such as 466.19 for bronchitis before he or she actually determines the underlying cause of the condition. Therefore, these analyses would not include visits that include diagnoses that are related to asthma. These analyses focused on diagnosed asthma.

Finally, our cost estimates are based on the Marketscan Medicaid data set for 2011. This data set contains claims data for Medicaid enrollees in 11 unidentified participating states. Although the average cost for an asthma-related ED visit (and subsequent hospitalizations) is $433 for these states, individual states may have higher or lower asthma treatment costs because Medicaid payment rates vary by state and provider type (21). According to a 2009 report by New York State, the average cost to Medicaid managed care enrollees for an asthma-related ED visit in 2007 was $243 (22). Therefore, we believe that the estimate taken from the 2011 Marketscan data is not out of reason. Furthermore, this estimate accounts for only the actual reimbursement by Medicaid for the ED visit, which is typically lower than reimbursements by other payers as well as lower than actual costs.

This analysis provides state-based estimates of costs for pediatric ED visits and brings attention to how much states are spending for this preventable condition. The CMS rule change described in this article may help states reduce costs of asthma treatment and at the same time reduce the severity of this condition through better management. Reduced costs for treating asthma may improve the overall quality of life and productivity for children and adolescents by improving asthma symptoms and reducing the number of school days missed due to asthma. We believe that the information presented here, despite its limitations, can be useful to policy makers in assessing options for decreasing Medicaid costs of asthma.
